# Relative maternal protection against type 1 diabetes: A combined analysis of 5 observational studies

**DOI:** 10.1210/clinem/dgag103

**Published:** 2026-03-10

**Authors:** Lowri A Allen, Peter N Taylor, Annelie Carlsson, Diane P Fraser, William A Hagopian, Emma Hedlund, Anita V Hill, Angus G Jones, Johnny Ludvigsson, Georgina L Mortimer, Suna Onengut-Gumuscu, Maria J Redondo, Stephen S Rich, Claire L Williams, Kathleen M Gillespie, Colin M Dayan, Richard A Oram

**Affiliations:** Diabetes Research Group, Cardiff University, University Hospital of Wales, Cardiff CF14 4XN, UK; Diabetes Research Group, Cardiff University, University Hospital of Wales, Cardiff CF14 4XN, UK; Department of Clinical Sciences, Lund University, Skånes University Hospital, SE-221 84 Lund, Sweden; Department of Clinical and Biomedical Sciences, University of Exeter Medical School, St Luke’s Campus, Exeter EX1 2LU, UK; NIHR Exeter Biomedical Research Centre, Exeter Centre of Excellence in Diabetes, University of Exeter Medical School, Royal Devon & Exeter Hospital (RILD Building), Exeter EX2 5DW, UK; Department of Pediatrics, Indiana University School of Medicine, Indianapolis, IN 46202, USA; Department of Clinical Sciences, Lund University, Skånes University Hospital, SE-221 84 Lund, Sweden; Kristianstad Central Hospital, SE-291 85 Kristianstad, Sweden; NIHR Exeter Biomedical Research Centre, Exeter Centre of Excellence in Diabetes, University of Exeter Medical School, Royal Devon & Exeter Hospital (RILD Building), Exeter EX2 5DW, UK; NIHR Exeter Clinical Research Facility, Royal Devon University Healthcare NHS Foundation Trust, Royal Devon & Exeter Hospital (RILD Building), Exeter EX2 5DW, UK; Department of Clinical and Biomedical Sciences, University of Exeter Medical School, St Luke’s Campus, Exeter EX1 2LU, UK; Endocrinology and Diabetes, Royal Devon University Healthcare NHS Foundation Trust, Royal Devon & Exeter Hospital, Exeter EX2 5DW, UK; Crown Princess Victoria Children’s Hospital, 581 85 Linköping, Sweden; Department of Biomedical and Clinical Sciences, Linköping University, 581 83 Linköping, Sweden; Translational Health Sciences, Bristol Medical School, University of Bristol, Bristol BS10 5NB, UK; Department of Genome Sciences, University of Virginia, Charlottesville, VA 22903, USA; Pediatric Diabetes and Endocrinology, Texas Children's Hospital, Houston, TX 77030, USA; Department of Pediatrics, Baylor College of Medicine, Houston, TX 77030, USA; Department of Genome Sciences, University of Virginia, Charlottesville, VA 22903, USA; Translational Health Sciences, Bristol Medical School, University of Bristol, Bristol BS10 5NB, UK; Translational Health Sciences, Bristol Medical School, University of Bristol, Bristol BS10 5NB, UK; Diabetes Research Group, Cardiff University, University Hospital of Wales, Cardiff CF14 4XN, UK; Department of Clinical and Biomedical Sciences, University of Exeter Medical School, St Luke’s Campus, Exeter EX1 2LU, UK; Endocrinology and Diabetes, Royal Devon University Healthcare NHS Foundation Trust, Royal Devon & Exeter Hospital, Exeter EX2 5DW, UK

**Keywords:** familial, family, maternal, offspring, paternal, protection, transmission, type 1 diabetes

## Abstract

**Context:**

Maternal (vs paternal) type 1 diabetes is associated with a relative reduction in type 1 diabetes risk in offspring during early life.

**Objective:**

To determine whether this effect extends into later life. To clarify the importance of intrauterine exposure to maternal type 1 diabetes, and baseline genetic susceptibility in this context.

**Methods:**

We compared the proportion of individuals with type 1 diabetes diagnosed aged 0 to 88 years of age with affected mothers and fathers across 5 observational studies (n = 11 475), and used random-effects meta-analyses to generate overall effect estimates. We examined this by age at diagnosis, and timing of parental diagnosis relative to offspring birth. We compared the type 1 diabetes genetic risk score (T1D-GRS2) of individuals with affected mothers and fathers.

**Results:**

Almost half as many individuals with type 1 diabetes had an affected mother vs father (odds ratio [OR], 0.55; 95% CI, 0.48-0.64; *P* < .0001). A lower proportion of individuals with affected mothers than fathers was apparent even among individuals diagnosed as adults (>18 years) (OR, 0.63; 95% CI, 0.43-0.91; *P* = .01). The lower proportion of individuals with maternal vs paternal type 1 diabetes was only observed if maternal diagnosis preceded offspring birth (OR, 0.51; 95% CI, 0.37-0.70; *P* < .001 vs OR 0.97; 95% CI, 0.69-1.38; *P* = .87 after birth). T1D-GRS2 was similar between individuals with affected mothers and fathers (*P* = .25).

**Conclusion:**

Our analyses suggest intrauterine exposure to maternal type 1 diabetes is associated with long-lasting relative protection against offspring type 1 diabetes, which is independent of genetic susceptibility as measured by T1D-GRS2.

Type 1 diabetes is a chronic autoimmune disease resulting from a combination of genetic predisposition and environmental exposures. Individuals with a family history are 8 to 15 times more susceptible than the background population (1-3). The risk is higher if the affected relative is the father vs mother (4).

Almost 40 years ago, Warram et al reported, in a cohort of 289 individuals with type 1 diabetes, the risk of type 1 diabetes was approximately 4 times higher in offspring of men vs women (6.1 ± 1.8% vs 1.3 ± 0.9%) (5). Larger prospective studies following offspring of individuals with type 1 diabetes have since replicated the finding of fewer offspring of women vs men developing the disease during early life (6-8). This is consistent with studies reporting fewer individuals with type 1 diabetes have affected mothers than fathers (9-13). Detailed descriptions of these studies are published elsewhere (4). Together, they demonstrate type 1 diabetes is approximately half as common during early life among offspring of women vs men with type 1 diabetes (4, 6, 7, 9-14). Comparison of the risk against that in individuals with affected siblings confirms this is due to a lower risk than expected among offspring of affected mothers (9, 10, 12). Maternal protection is only relative, as the risk remains higher than in the background population (2%-3% lifetime risk vs 0.3%) (4, 13).

To date, studies have typically followed offspring to age 20 to 30 years (4, 6, 7, 9-14). A recent study by Wei et al reported relative maternal protection in offspring diagnosed with type 1 diabetes in childhood (<18 years) and adulthood (>18 years) but did not include offspring diagnosed beyond age 30 years (14). Tillil et al published the only study including offspring diagnosed beyond age 30 years, but included few individuals diagnosed as older adults (n = 138 > 25 years, n = 23 > 40 years.) (10) It remains unclear whether maternal protection simply delays disease onset, which could still be beneficial given later onset is associated with improved outcomes (15), or whether it represents a long-term effect persisting into later life.

A range of potential mechanisms have been considered and can be broadly grouped as genetic and environmental (4). One approach to disentangling genetic from environmental contributions is to examine whether protection occurs only when mothers are diagnosed before pregnancy, which would implicate intrauterine exposure as an important factor. A few small studies have explored this, with conflicting results (6, 12, 13, 15, 16).

This study used data from 5 observational studies of individuals with type 1 diabetes (n = 11 475). Our aims were first to determine whether any relative protection associated with maternal type 1 diabetes is confined to early life. Second, we wanted to clarify whether differences in parental rates of type 1 diabetes among individuals with type 1 diabetes depend on parental disease being diagnosed before offspring birth, as suggested by some reports. Finally, we wanted to determine if relative maternal protection depends on differences in type 1 diabetes genetic susceptibility (measured by type 1 diabetes genetic risk score [T1D-GRS2]) between participants with affected mothers and fathers.

## Materials and methods

This study used data from 5 observational studies of individuals with type 1 diabetes that collected family history and genetics data in the form of T1D-GRS2.

The Barts Oxford Family Study (BOX) has recruited individuals with newly diagnosed type 1 diabetes before age 21 years since 1985 in the Oxfordshire Health Authority Region, UK (17, 18). Participants and relatives (with and without type 1 diabetes) have been prospectively followed. Similarly, Better Diabetes Diagnosis (BDD) and StartRight are also observational studies of individuals with newly diagnosed diabetes (BDD; individuals diagnosed with type 1 diabetes before age 18 years in Sweden since 2005 (19), StartRight; individuals with newly diagnosed diabetes ≥17 years in the United Kingdom between 2015 and 2020). The Type 1 Diabetes Genetic Consortium (T1DGC) “families dataset” (20) and TrialNet Pathway to Prevention (TrialNet PTP) (21) represent 2 additional sources of family and T1D-GRS2 data collected from individuals with type 1 diabetes, but were designed to overrepresent individuals with affected relatives. The former is an international study of predominantly affected sibling pairs with type 1 diabetes conducted between 2004 and 2009. The latter has recruited and followed first-degree relatives of individuals with type 1 diabetes positive for at least 1 type 1 diabetes-related autoantibody since 2000. Detailed study descriptions are provided (Table 1).

**Table 1:** Summary of the studies from which participants were included

Study	Number of participants meeting eligibility criteria for inclusion in this study	Age at diagnosis of type 1 diabetes	Recruitment Area	Recruitment period	Type 1 diabetes at enrollment?
BOX (17, 18)	3040	<21 years Median 10 years (IQR 6, 13 years)	Oxfordshire Health Authority Region, UK	Since 1985	Yes (newly diagnosed). Diagnosis of type 1 diabetes based on WHO criteria and clinical requirement for insulin treatment.
BDD (19)	3896	<18 years Median 10 years (IQR 6, 14 years)	Sweden (nationwide)	Since 2005	Yes (newly diagnosed) since 2005. Since 2011 estimated to have captured >95% of new diagnoses of type 1 diabetes younger than age 18 years. To establish diabetes classification, the study included analyses of HLA-DQ genotypes, autoantibodies and levels of C-peptide at first recruitment.
TrialNet PTP (21, 22)	1316	0-55 years Median 12 years (IQR 8, 16 years)	International, multicenter Participating countries include the United States of America, Canada, United Kingdom, Germany, Italy, Sweden, Finland, Australia, and New Zealand	Since 2000	No. Recruitment of first-degree relatives of individuals with type 1 diabetes found to be positive for at least one type 1 diabetes associated autoantibody. Progression to type 1 diabetes (on the basis of the results of oral glucose tolerance test, HbA1c level, and antibody testing results) was recorded.
T1DGC “Families Dataset” (20)	2662	0-32 years Median 7 years (IQR 4, 12 years)	International, multicenter. Data collected across 4 regional networks: Asia-Pacific (participating countries Australia, India, Malaysia, New Zealand, Philippines, Singapore, Thailand) Europe (Austria, Belgium, Cameroon, Czech Republic, Denmark, Estonia, Finland, Germany, Greece, Hungary, Israel, Italy, Latvia, Lithuania, Netherlands, Poland, Portugal, Romania, Russia, Slovenia, Spain, Sweden, Switzerland, Turkey) North America (57 data collection sites in the United States of America) United Kingdom Network (48 data collection sites in the United Kingdom)	2004-2009	Yes. Recruitment of predominantly affected sibling pairs and a smaller group of parent-child trios to generate a “families dataset” within the T1DGC.
StartRight (23, 24).	561	17-88 years Median 35 years (IQR 27, 49 years)	55 UK sites	2015-2020	Recruited participants with newly diagnosed diabetes (<12 months’ duration). Type of diabetes recorded and validated using autoantibody testing and clinical information.

Descriptions of the individual studies contributing to our analyses are provided.

Abbreviations: BDD, Better Diabetes Diagnosis; BOX, Barts Oxford Family Study; HbA1c, glycated hemoglobin; IQR, interquartile range; WHO, World Health Organization.

Inclusion criteria for this study were type 1 diabetes and the availability of family history. Individuals were excluded if their relation to an affected relative was uncertain. All individuals in BOX (n = 3040), BDD (n = 3896), and T1DGC “families dataset” (n = 2662) were eligible. In TrialNet PTP, 1316 individuals progressed to type 1 diabetes and were eligible. Of 1800 individuals recruited to StartRight, 561 had type 1 diabetes and were eligible. A total of 11 475 participants were ultimately included (Fig. S1 (25)).

### Data collection

Family history was obtained through questioning of participants and/or relatives at recruitment and during follow up. Parents with type 1 diabetes were recorded in all studies except StartRight, in which relatives with diabetes were recorded, but diabetes type was not. Consistent with our prespecified analysis plan, treatment with insulin only was used as a surrogate for type 1 diabetes in relatives in StartRight (26). Results using more restrictive definitions were similar (Table S1 (25)).

Data regarding the timing of parents’ diagnoses relative to offspring birth were available in BOX and TrialNet PTP. Data regarding affected siblings were available for all studies except BDD.

T1D-GRS2 is a validated measure of polygenic susceptibility to type 1 diabetes. T1D-GRS2 was generated as previously described in T1DGC and StartRight (23, 27). T1D-GRS2 was generated in BDD and BOX with a custom single nucleotide polymorphism (SNP) genotyping panel developed by Dr. Hagopian with LGC Genomics using KASP genotyping. T1D-GRS2 in TrialNet PTP was generated as previously described (27), using genotyping from an Illumina Infinium T1DExomeChip SNP array, imputed to the TOPMed reference panel (supplementary text 12 (3)).

T1D-GRS2 was available for all BDD, TrialNet PTP, and T1DGC participants. In StartRight, T1D-GRS2 was available for 511 (95%) participants. In BOX, initiated in 1985, T1D-GRS2 was available for 1388 (46%) individuals. Prospective and retrospective DNA sampling commenced in 1998 but fewer existing families provided samples. Age at diagnosis and parental type 1 diabetes were comparable between individuals who did and did not provide samples (median diagnosis age 10.5 vs 10.1 years, 2.7% affected mothers vs 2.9%, 5.0% affected fathers vs 5.0%).

### Statistical analysis

Analyses were undertaken separately within each study initially. Because of the heterogeneity of studies, unadjusted random effects meta-analyses were used to derive effect estimates across studies.

The proportion of missing data is described. Because missing data were minimal (<1%) for most analyses, complete case analyses were undertaken. Eleven percent of cases had missing T1D-GRS2, predominantly because of missing data in BOX. BOX was attributed lower weighting accordingly. BOX results were consistent with other studies.

The proportion of individuals with type 1 diabetes with affected mothers and fathers are presented as frequencies (percentages) for individual studies. Participants with both parents affected (0.26% overall, Table S2 (25)) were included in both counts. Because of sibling data being unavailable for BDD, and differences in study design resulting in overrepresentation of affected siblings in TrialNet PTP and T1DGC (Table S3 (25)), individuals with and without affected siblings were not treated differently.

The ratio of the odds of an affected mother to the odds of an affected father, are presented as odds ratios (ORs) and 95% CIs for each study. Random effects meta-analysis was used to derive an overall OR across studies. The same methods were used to generate results for individuals diagnosed ≤18 years and >18 years.

The mean difference in age and T1D-GRS2 were compared between those with affected mothers vs fathers. Standardized mean differences across studies were derived from random effects meta-analyses.

Using data from BOX and TrialNet PTP, the proportion of individuals with an affected mother vs father was compared between those whose parents were diagnosed before and after birth.

### Additional analyses

A limitation of our study is that complete data regarding offspring who did not develop type 1 diabetes were not available for all cohorts. Reporting the proportion of individuals with type 1 diabetes with affected mothers vs fathers is consistent with the approach of several publications (9-13). These publications generated results similar to those obtained from prospective studies including all offspring of men and women with type 1 diabetes (6-8). However, an observed difference in parental rates of type 1 diabetes could theoretically be explainable by: (1) an excess of males relative to females with type 1 diabetes, (2) more offspring being born to men than women with type 1 diabetes, and (3) an excess risk of type 1 diabetes among offspring of affected men, as opposed to relative maternal protection. We undertook a series of analyses to address each possibility.

1. Type 1 diabetes is relatively more common in males than females (28-30). This study was not designed to adjust for this. However, we examined the sex distribution of participants. Published literature indicates that the recognized male preponderance of type 1 diabetes is driven by an excess of male diagnoses at older ages (28). Depending on the population studied, the ratio of male to females among individuals diagnosed after age 15 years is 1.3 to 2.2. However, prior to age 15 years, the sex distribution is relatively equal (28-30). In a Swedish nationwide study, a significant male bias was observed among individuals diagnosed ages 15 to 19 and 20 to 24 years (ratios 1.59, 2.08, respectively) but not 10 to 14 years (0.94) (31). We therefore undertook a sensitivity analysis restricted to individuals with parents diagnosed before age 15 years.
2. Data regarding the total number of children born to men and women with type 1 diabetes were only available in BOX. An unpaired Student *t*-test was used to compare the mean number of children born to men and women with type 1 diabetes.
3. BOX was the only study to record complete sibling data *and* not bias recruitment toward individuals with affected siblings. We described the proportion of participants with affected siblings compared with affected mothers and fathers.

All statistical analyses were performed using STATAv17.0. *P* < .05 was deemed significant.

Ethical approval was obtained before establishing each individual study (17-19, 21, 23, 24, 32). This study fell within the remit of original approvals. All participants gave informed consent, with specific assent procedures for children, and oversight from at least 1 ethical review board (minimum 1 per country for international studies), in accordance with the Declaration of Helsinki).

## Results

A total of 11 475 individuals with type 1 diabetes were eligible for inclusion. Participants were diagnosed at ages 0 to 88 years (median 10, IQR 6-14). A total of 1052 (9.2% of participants) individuals were diagnosed after age 18 years, 648 (5.6%) > 25 years, 515 (4.5%) > 30 years, 292 (2.5%) > 40 years (Table S4 (25)).

**Figure 1:**
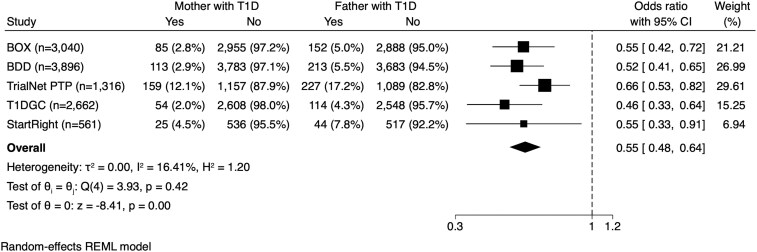
Meta-analysis of odds of having a mother with type 1 diabetes compared with the odds of having a father with type 1 diabetes among individuals with type 1 diabetes. The number and proportion (%) of individuals with affected mothers and fathers are shown for each individual study. Unadjusted odds ratios are shown for the odds of individuals diagnosed with type 1 diabetes having an affected mother vs father. The odds ratios for each individual study are shown, as well as the overall odds ratio as derived from a random effects meta-analysis. *P* < 0.0001. Abbreviation: T1D, Type 1 diabetes.

Fewer individuals with type 1 diabetes had an affected mother vs father (OR, 0.55; 95% CI, 0.48-0.64; *P* < .0001) (Fig. 1, Tables S2 and S3 (25)). Results were comparable across studies. Specifically, results were similar in StartRight (individuals diagnosed with type 1 diabetes as adults (≥17 years) (4.5% vs 7.8%; OR, 0.55;0.33-0.91; *P* = .02) and studies of individuals diagnosed in early life (BOX (<21 years), 2.8% vs 5.0%; OR, 0.55; 0.42-0.72); *P* < .0001; and BDD (<18 years) 2.9% vs 5.5%; OR, 0.52; 0.410.65; *P* < .0001) (Fig. 1).

**Figure 2:**
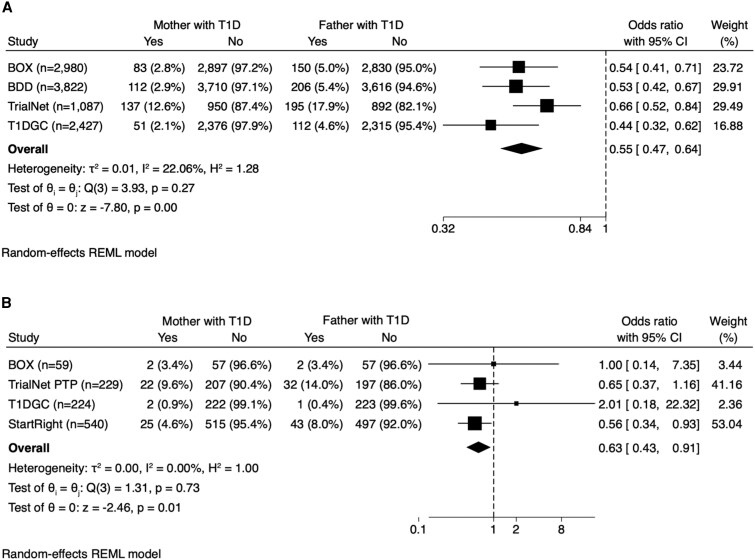
Meta-analyses of odds of having a mother with type 1 diabetes compared with a father with type 1 diabetes in individuals with type 1 diabetes, divided according to age at diagnosis. (A) Individuals diagnosed with type 1 diabetes ≤18 years of age. The number and proportion (%) of individuals with affected mothers and fathers are shown for individuals diagnosed with type 1 diabetes at or before the age of 18 years. Unadjusted odds ratios are shown for the odds of these individuals having an affected mother vs father. The odds ratios for each individual study are shown, as well as the overall odds ratio as derived from a random effects meta-analysis. *P* < .0001. StartRight contributed no mothers and just 1 father to this analysis and therefore was dropped from the analysis. As expected, the overall result was the same regardless of whether StartRight was included (OR, 0.55; 95% CI, 0.47-0.64). (B) Individuals diagnosed with type 1 diabetes >18 years of age. The number and proportion (%) of individuals with affected mothers and fathers are shown for individuals diagnosed with type 1 diabetes after age 18 years. Unadjusted odds ratios are shown for the odds of these individuals having an affected mother vs father. The odds ratios for each individual study are shown, as well as the overall odds ratio as derived from a random effects meta-analysis. *P* = .01. Abbreviation: T1D, Type 1 diabetes. Missing data regarding age at diagnosis: BOX n = 1, BDD n = 74, TrialNet PTP n = 0, T1DGC n = 11, StartRight n = 0 (0.7% of all cases in study affected by missing data).

Significantly fewer individuals had an affected mother compared with father in meta-analyses of participants diagnosed >18 years (OR, 0.63; 0.43-0.91; *P* = .01) and ≤18 years (OR, 0.55; 0.47-0.64; *P* < .0001) (Fig. 2). Analyses using cutoffs between 13 and 19 years (Table S5 (25)) yielded similar results.

**Figure 3:**
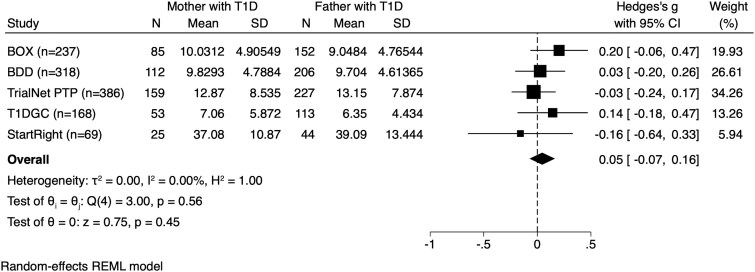
Comparison of age at onset of type 1 diabetes between individuals with mothers and fathers with type 1 diabetes. Hedges's g standardized mean difference in age at diagnosis (in years) between individuals with type 1 diabetes and an affected mother compared with father. The results are shown for the individual studies, and an overall estimate of effect as derived from random effects meta-analysis. Complete case analysis was performed. *P* = .45. Abbreviation: T1D, Type 1 diabetes. Missing data regarding age at onset among individuals with parents with type 1 diabetes: BOX n = 0, BDD n = 8, TrialNet PTP n = 0, T1DGC n = 2, StartRight n = 0 (total n = 10, 0.8%).

Age at diagnosis was similar among participants with affected mothers and fathers (overall Hedges's g standardized mean difference 0.05 years; 95% CI, −0.07 to 0.16; *P* = .45) (Fig. 3).

### Relative maternal protection

To ascertain whether our findings indicate relative maternal protection against offspring type 1 diabetes, we sought to exclude alternative explanations.

1. Is the effect due to type 1 diabetes being more common in males than females?

The ratio of male to female participants was 1.15 (Table S6 (25)). This is similar to published results from comparable populations (29). Assuming the sex distribution of type 1 diabetes is stable across generations, as described by Gale et al (28), this slight male excess is too small to account for the magnitude of the difference in maternal vs paternal disease in our study. Furthermore, restricting analysis to individuals with parents diagnosed before age 15 years, among whom no significant sex bias is expected, we still found a lower proportion of individuals with affected mothers (OR, 0.69; 0.50, 0.95; *P* = .02) (Fig. S2 (25)).

2. Is it due to more offspring being born to men than women with type 1 diabetes?

In BOX, the mean number of children born to men and women with type 1 diabetes was similar (mean 2.4 children for both, *P* = .83) (Table S7 (23)).

3. Is it due to an excess risk of type 1 diabetes among offspring of men with type 1 diabetes?

In BOX (n = 3040), the proportion of participants with affected siblings and fathers was comparable (n = 168 [5.5%], n = 152 [5.0%]). The proportion with an affected mother was lower (n = 85 [2.8%.]) This is in keeping with offspring of affected mothers being at relatively lower risk, as opposed to excessive risk among offspring of affected fathers.

### Intrauterine exposure to maternal type 1 diabetes

**Figure 4:**
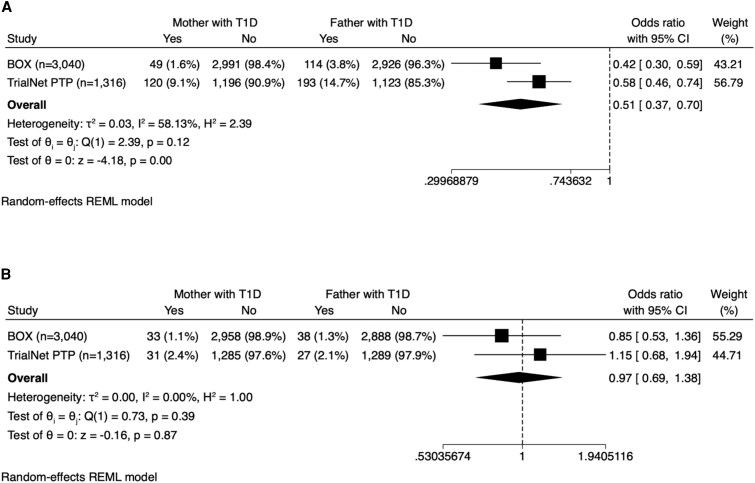
Meta-analyses of odds of having a mother with type 1 diabetes compared with a father with type 1 diabetes in individuals with type 1 diabetes, divided into subgroups according to whether parental diagnosis was made prior to or after the birth of the offspring. (A) Parental diagnosis before the birth of the offspring. The number and proportion (%) of individuals with affected mothers and fathers are shown for when analysis is confined to individuals with parents diagnosed before offspring birth. Unadjusted odds ratios shown for the odds of individuals with type 1 diabetes having an affected mother vs father. The odds ratios for each individual study are shown, as well as the overall odds ratio as derived from a random effects meta-analysis. These data were only available from BOX and TrialNet PTP. *P* < .001. Abbreviation: T1D, Type 1 diabetes. (B) Parental diagnosis after the birth of the offspring. The number and proportion (as % of those at risk, after excluding those with a mother/father diagnosed before their birth as appropriate) of individuals with affected mothers and fathers are shown for when analysis is confined to individuals with parents diagnosed after offspring birth. Unadjusted odds ratios are shown for the odds of individuals with type 1 diabetes having an affected father vs mother. The odds ratios for each individual study are shown, as well as the overall odds ratio as derived from a random effects meta-analysis. These data were only available from BOX and TrialNet PTP. *P* = .87. Abbreviation: T1D, Type 1 diabetes. Missing data regarding timing of parental diagnosis: BOX fathers n = 0, mothers n = 3, TrialNet PTP fathers n = 7, mothers n = 8.

Among participants with parents diagnosed before their birth, significantly fewer had an affected mother than father (OR, 0.51; 0.37-0.70; *P* < .001) (Fig. 4A). A lower proportion of individuals with an affected mother was not apparent among individuals with parents diagnosed after their birth (OR, 0.97; 0.69-1.38; *P* = .87 (Fig. 4B).

Parents diagnosed before offspring birth are inevitably younger at diagnosis than parents diagnosed after offspring birth. Earlier disease onset is associated with increased genetic susceptibility and increased transmission to offspring. We therefore expected more participants to have parents diagnosed before vs after birth. This is what we observed for fathers (Table 2) (BOX: n = 38 [1.3%] after birth, n = 114 [3.8%] prebirth, TrialNet PTP n = 27 [2.1%] after birth, n = 193 [14.7%] prebirth). This was less apparent for mothers because of fewer than expected participants having mothers diagnosed before their birth (BOX: n = 33 [1.1%] after birth, n = 49 [1.6%] prebirth, TrialNet PTP n = 31 [2.4%] after birth, n = 120 [9.1%] prebirth). These findings suggest intrauterine exposure to maternal type 1 diabetes is important.

**Table 2:** Comparison of proportion of individuals with an affected mother vs father, divided into subgroups according to whether parental diagnosis was made before or after the birth of the offspring

Study	Number (%) of individuals with mother diagnosed prior to birth	Number (%) of individuals with father diagnosed prior to their birth	Odds ratio (95% CI)	*P* value	Number (%) of individuals with mother diagnosed after their birth	Number (%) of individuals with father diagnosed after their birth	Odds ratio (95% CI)	*P* value
BOX (n = 3040)	49 (1.6%)	114 (3.8%)	0.42 (0.30-0.59)	<.0001	33 (1.1%)	38 (1.3%)	0.85 (0.53-1.36)	.49
TrialNet PTP (n = 1316)	120 (9.1%)	193 (14.7%)	0.58 (0.46-0.74)	.0003	31 (2.4%)	27 (2.1%)	1.15 (0.68-1.94)	.14
Pooled estimate as derived from random effects meta-analysis			0.51 (0.37-0.70)	.001			0.97 (0.69-1.38)	.12

Data regarding timing of parental diagnosis were only available in Barts Oxford Family Study (BOX) and TrialNet Pathway to Prevention. For these studies, the number and proportion (%) of individuals with an affected mother vs father are shown according to whether the parents were diagnosed before or after offspring birth. Data regarding the timing of diabetes diagnosis were not available for 3 mothers in BOX, and for 8 mothers and 7 fathers in TrialNet Pathway to Prevention.

### Genetic susceptibility

**Figure 5:**
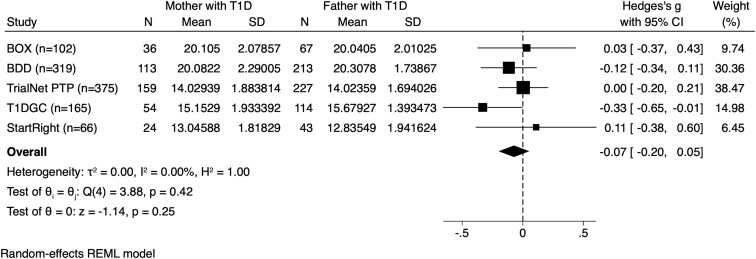
Comparison of type 1 diabetes genetic risk score (T1D-GRS2) between individuals with mothers and fathers with type 1 diabetes. Hedges's g standardized mean difference in type 1 diabetes genetic risk score (T1D-GRS2) for individuals with an affected mother compared with an affected father. The results are shown for the individual studies, alongside an overall estimate of effect as derived from random effects meta-analysis. Complete case analysis was performed. *P* = 0·25. Abbreviation: T1D, Type 1 diabetes. Missing data regarding T1D-GRS2 amongst individuals with parents with type 1 diabetes: BOX n = 134, BDD n = 0, TrialNet PTP n = 0, T1DGC n = 0, StartRight n = 2 (total n = 136, 11%).

T1D-GRS2 was similar between participants with affected mothers vs fathers (overall Hedges's g standardized mean difference −0.07; 95% CI, −0.20 to 0.05; *P* = .25) (Fig. 5). T1D-GRS2 was lower in StartRight than other studies, in keeping with the older age at diagnosis of participants (Fig. 5). Results of a sensitivity analysis excluding StartRight were comparable (overall Hedges's g standardized mean difference −0.09; 95% CI, −0.22 to 0.05; *P* = .20 (Fig. S3 (25)).

## Discussion

In this large study (n = 11 475) incorporating meta-analyses across 5 studies, we confirm that approximately half as many individuals with type 1 diabetes have affected mothers than fathers (Fig. 1). We report for the first time that this is evident even among individuals diagnosed in later adulthood (Fig. 2), and demonstrate no significant difference in age at diagnosis between individuals with affected mothers and fathers (Fig. 3). We only observed a lower proportion of participants with affected mothers when maternal diagnosis was before offspring birth, resolving earlier uncertainty regarding this (Fig. 4). Through exploratory analyses, we demonstrate our findings to be in keeping with relative maternal protection against offspring type 1 diabetes. Finally, we show for the first time, that genetic susceptibility (T1D-GRS2) was similar in individuals with affected mothers and fathers (Fig. 5), suggesting that the observed difference is not explained by inherited genetic risk. Together, our data provide novel insights into relative maternal protection, demonstrating this is an enduring phenomenon that follows intrauterine exposure to maternal type 1 diabetes, which is independent of inherited type 1 diabetes genetic susceptibility (measured by T1D-GRS2), and continues to protect offspring into adulthood.

The study population was confined to individuals with type 1 diabetes. Offspring without type 1 diabetes were not included. Therefore, to be confident our observation of fewer participants having affected mothers than fathers was indicative of relative maternal protection, we sought to exclude alternative explanations. First, type 1 diabetes is relatively more common in males, and this could account for fewer participants having affected mothers than fathers. Consistent with published results from comparable populations, we observed a slight male bias among participants (male:female ratio 1:15; Table S6 (25)). This is insufficient to account for the magnitude of the difference in the proportion of affected mothers and fathers. Furthermore, even after restricting analysis to individuals with parents diagnosed before age 15 years (among whom no significant sex bias is expected (28-31)), we still found fewer individuals had affected mothers than fathers (Fig. S2 (25)). These findings suggest our results are not due to an excess of males with type 1 diabetes.

An alternative possibility is that more children are born to men than women with type 1 diabetes. For example, it is well documented that maternal type 1 diabetes is associated with an increased risk of complications for both mother and baby, and that pregnancy can make the management of type 1 diabetes significantly more challenging (eg, due to pregnancy being a state of relative insulin resistance) (33). It is therefore possible that some women with type 1 diabetes may choose to have fewer pregnancies or even avoid pregnancy altogether because of concerns about the associated risks. However, we report that in BOX, men and women with type 1 diabetes had a similar number of children (mean 2.4 for both; Table 7 (25)). This is consistent with findings from a Danish cohort, which reported fewer individuals with type 1 diabetes had affected mothers than fathers despite men and women with type 1 diabetes having a similar number of children (2.16 vs 1.96) (12). Taken together, our analyses suggest that our results indicate a true difference in type 1 diabetes risk between offspring of affected mothers and fathers.

BOX was the only study to collect complete sibling data and where participant recruitment was not biased toward individuals with affected siblings. In BOX, a similar proportion of participants had affected siblings and fathers (5.5% and 5.0% respectively), but fewer had affected mothers (2.8%). This is consistent with findings of published studies (9, 10, 12), and in the context of our other results, supports the phenomenon of relative maternal protection against offspring type 1 diabetes, rather than an excess of paternal transmission.

We confirm that relative maternal protection is only observed if the mother already has diabetes before offspring birth. Women diagnosed before pregnancy will inevitably be younger at diagnosis than mothers diagnosed after pregnancy. Therefore, maternal age at diagnosis could be an important confounder. This study was not designed to directly examine this. Harjutsalo et al reported that among offspring of mothers diagnosed with type 1 diabetes aged 0 to 39 years, relative maternal protection was greater if mothers were diagnosed before age 10 years (15). Because a younger age at diagnosis is associated with high-risk type 1 diabetes susceptibility genes (34, 35), it is plausible that if maternal protection is driven by reduced maternal transmission of these genes, the impact might be greatest among offspring of parents diagnosed earlier in life. However, our study contained approximately 3 times as many children born to mothers with type 1 diabetes (n = 436 vs 150), and we found that type 1 diabetes genetic susceptibility measured by T1D-GRS2 was similar between participants with affected mothers and fathers. We acknowledge that T1D-GRS2 is an imperfect measure of inherited genetic susceptibility, based on 67 specific SNPs, but in the absence of sufficient power to study individual genetic variants, our results suggest that aggregate genetic susceptibility to type 1 diabetes was not significantly different in individuals with type 1 diabetes with affected mothers vs fathers. Our results do not exclude the possibility of a genetic mechanism not detectable using T1D-GRS2, including a difference in rare variant inheritance between offspring of men and women with type 1 diabetes, nor the possibility of genetic imprinting, whereby the expression of inherited genes differs according to the parent from whom they are inherited. However, neither (as sole mechanisms) would account for our observation that maternal protection is only apparent when maternal disease is diagnosed before offspring birth. Furthermore, 2 large studies (including a genome-wide association study) have concluded genetic imprinting is unlikely to significantly contribute to type 1 diabetes susceptibility in offspring of affected men and women (36, 37). Larger studies are required to definitively examine the interaction between timing of parental diagnosis and parental age at diagnosis.

Potentially important intrauterine exposures may include maternal hyperglycemia, maternal islet antibodies, exogenous insulin therapy, maternal antibodies to insulin, and maternal antibodies to enterovirus (a type 1 diabetes trigger) (4, 38-41). Toxicity from hyperglycemic exposure is deemed the principal driver for increased spontaneous abortions in women with type 1 diabetes (4, 42-44). Consequently, it is plausible that selective loss of fetuses exposed to severe hyperglycemia may contribute to relative maternal protection. This is difficult to study because many pregnancy losses occur early in pregnancy and go unrecorded. However, our data suggest the number of children born to men and women with type 1 diabetes is comparable (Table S7 (25)), consistent with a previous report (12).

For surviving offspring, intrauterine exposure to 1 or more of the exposures listed previously may impact offspring type 1 diabetes susceptibility by directly influencing the development and maturation of the pancreatic beta cells and/or the immune system. As an example, intrauterine exposure to hyperglycemia could plausibly stimulate beta-cell growth, development, and maturation, thereby reducing offspring type 1 diabetes susceptibility. However, there may be a threshold whereby exposure to very marked degrees of hyperglycemia may lead to beta-cell exhaustion and therefore an increased risk of type 1 diabetes (45). Either separately, or in concert, the transplacental transfer of maternal antibodies could directly induce immunotolerance against autoantigens and/or promote efficient clearance of autoreactive T cells (40). A more detailed description of potentially relevant intrauterine exposures and the effects they could plausibly exert on the development of the pancreas and/or immune system are provided elsewhere (4).

Intrauterine exposure to these different components of maternal type 1 diabetes could also have more indirect effects on offspring type 1 diabetes susceptibility (eg, via epigenetics). Epigenetic mechanisms have been documented to influence T-cell differentiation and maturation, insulin gene expression and beta cell function. and autoantigen expression (46). Such epigenetic changes can be detected even before the development of islet autoimmunity, suggesting a possible role for epigenetics in the pathogenesis of the disease (47). It is plausible that these epigenetic changes could be driven, at least in part, by intrauterine exposures, including potentially maternal type 1 diabetes. Knorr et al reported evidence of epigenetic changes in adolescent offspring of mothers with type 1 diabetes who had been exposed to hyperglycemia in utero (48). Larger studies are required to validate this finding. Future studies should consider that there may not be one sole mechanism through which these exposures influence type 1 diabetes susceptibility, and that some of these mechanisms could complement or interact with one another.

Insights into the relative importance of exposure to maternal hyperglycemia could be gained from comparing the type 1 diabetes risk of offspring of parents with different forms of diabetes. A systematic review published in 2019 reported that the risk of type 1 diabetes in offspring of mothers with gestational diabetes was higher than for mothers without diabetes, but lower than for mothers with type 1 diabetes (49). The risk of type 1 diabetes was not significantly different between offspring of mothers with type 2 diabetes and mothers without diabetes. However, maternal diabetes diagnoses were not consistently and well characterized, relatively few mothers had type 2 diabetes, and important potential confounders were not addressed. Large, robustly designed studies of offspring of parents with different forms of diabetes are required. Ultimately, studies that include measures of maternal glycemia at different time points during pregnancy are required to define the relationship between exposure to maternal hyperglycemia in different forms of maternal diabetes and offspring type 1 diabetes risk. Such studies should consider that birthweight may be either a confounder or an effect mediator in the relationship between exposure to maternal hyperglycemia and offspring type 1 diabetes risk (4).

Strengths of our study include the use of data from 5 large studies (n = 11 475), encompassing individuals diagnosed with type 1 diabetes between ages 0 and 88 years, over more than 3 decades, recruited from 4 continents. The heterogeneity of the population suggests our findings likely apply to a broad population of individuals with type 1 diabetes. The availability of T1D-GRS2 as a measure of genetic susceptibility incorporating HLA- and non-HLA genetic loci in these individuals provides important novel insights. Furthermore, our study includes more individuals diagnosed in later adulthood than published studies. The largest study including participants with adult-onset type 1 diabetes published by Wei et al (n = 3240) included only offspring diagnosed before age 30 years. Tillil et al published the only study including individuals diagnosed at older ages. Our study included more than 3 times as many offspring diagnosed beyond age 25 years (n = 648 [38 with an affected mother, 47 with an affected father] vs 138 (10). Larger studies of participants diagnosed in later life are required to confirm our findings.

The main limitation is that offspring without type 1 diabetes were not included, which restricts causal inference. Prospective lifelong follow up of all offspring of men and women with type 1 diabetes (regardless of diabetes status) would be optimal. Publications attempting this approach to date have only followed offspring into early adult life (4, 6, 7, 9-14). As registries of routinely collected clinical data mature, they will become invaluable resources for examining type 1 diabetes risk over the entire lifecourse of offspring. However, they are unlikely to routinely collect data such as T1D-GRS2.

Additional limitations include data regarding parental diagnoses timing only being available in selected studies. Another potential limitation was that family history was obtained by questioning participants and families. Parental type 1 diabetes could have been missed if an individual was estranged from a parent, but this is unlikely to disproportionately exclude more affected mothers than fathers. In StartRight, not specifically recording the type of diabetes in relatives posed a misclassification risk. However, use of increasingly restrictive definitions of parental type 1 diabetes did not impact our results, which were consistent with those from the studies that accurately recorded parental diagnoses. Despite its relatively low weighting in meta-analyses (reflecting n = 561), StartRight was important to include as the largest, best characterized study of adult-onset type 1 diabetes including family and T1D-GRS2 data.

In conclusion, our findings suggest that, independently of genetic susceptibility, intrauterine exposure to maternal type 1 diabetes is associated with relative protection against offspring type 1 diabetes that persists into adulthood. Further studies are required to identify the key exposures involved and to confirm these observations in larger prospective datasets.

A better understanding of how maternal type 1 diabetes results in a relative reduction in type 1 diabetes in offspring may provide opportunities to develop interventions that could reduce type 1 diabetes risk in predisposed individuals.

## Data Availability

Data can be requested directly from the data holders for each study and/or accessed via public repositories including dbGaP, EGA. The BOX Study Group comprises the study team based at the University of Bristol including Mrs Isabel Wilson, Mrs Rachel Aitken, Ms Ilana Kelland and Mrs Clare Megson; Local consultants in the BOX region are Drs Chitrabhanu Ballav, Atanu Dutta, and Michelle Russell-Taylor, Bucks Healthcare Trust, UK; Dr Rachel Besser, Oxford University Hospitals Trust UK, UK; Drs James Bursell and Shanthi Chandran, Milton Keynes University Hospital, UK, Dr Sejal Patel, Wexham Park Hospital, UK; Drs Anne Smith and Manohara Kenchaiah, Northampton General Hospital, UK; Dr Gomathi Margabanthu, Kettering General Hospital, UK; Drs Foteini Kavvoura and Chandan Yaliwal, Royal Berkshire Hospital, UK. The BDD Study is funded by The Swedish Child Diabetes Foundation. BDD comprises a national study group, Asst. Prof. Annelie Carlsson, Prof. Helena Elding Larsson. and Prof. Åke Lernmark, Department of Clinical Sciences, Lund University, Asst. Prof. Auste Pundziute Lyckå, and Asst. Prof. Gun Forsander, Department of Paediatrics, Institute for Clinical Science, Sahlgrenska Academy, University of Gothenburg, MD Anna EK, Asst. Prof. Martina Persson, Department of Medicine, Clinical Epidemiology, KI, Prof. Claude Marcus, KI, Prof Johnny Ludvigsson and Asst. Prof. Karin Åkesson, Department of Biomedical and Clinical Science, Linköping University, Linköping, Sweden. BMA Qefsere Brahim and Marlena Maziarz have worked with the BDD-data set. The Type 1 Diabetes TrialNet Study Group is a clinical trials network currently funded by the 10.13039/100000002National Institutes of Health (NIH) through the 10.13039/100000062National Institute of Diabetes and Digestive and Kidney Diseases, the 10.13039/100000060National Institute of Allergy and Infectious Diseases, and The 10.13039/100009633Eunice Kennedy Shriver National Institute of Child Health and Human Development, through the cooperative agreements U01 DK061010, U01 DK061034, U01 DK061042, U01 DK061058, U01 DK085461, U01 DK085465, U01 DK085466, U01 DK085476, U01 DK085499, U01 DK085509, U01 DK103180, U01 DK103153, U01 DK103266, U01 DK103282, U01 DK106984, U01 DK106994, U01 DK107013, U01 DK107014, U01 DK106993, UC4 DK117009, and the JDRF. NIH NIDDK R01DK121843 funded M.J.R., S.O.G., S.S.R., and R.A.O., as well as genotyping, imputation, and genetic score calculations for TrialNet samples. The Type 1 Diabetes Genetics Consortium, is a collaborative clinical study sponsored by the 10.13039/100000062National Institute of Diabetes and Digestive and Kidney Diseases (NIDDK), 10.13039/100000060National Institute of Allergy and Infectious Diseases (NIAID), 10.13039/100000051National Human Genome Research Institute (NHGRI), 10.13039/100009633National Institute of Child Health and Human Development (NICHD), and Juvenile Diabetes Research Foundation International (JDRF) and supported by U01 DK062418. The StartRight study was funded by 10.13039/501100000361Diabetes UK (17/0005624) and the 10.13039/501100000272National Institute for Health and Care Research (NIHR; CS-2015-15-018). Genotyping for generation of the T1D GRS was supported by the 10.13039/501100001648European Foundation for the Study of Diabetes (2016 Rising Star Fellowship). The study was supported by the 10.13039/501100013373NIHR Exeter Biomedical Research Centre and NIHR Exeter Clinical Research Facility. The StartRight Study Group is led & coordinated by the study team in Exeter, comprising Prof Angus Jones, Mrs Anita Hill, Prof Timothy McDonald, Prof Andrew Hattersley, Prof Beverley Shields, Mr Robert Bolt, Mr Peter Tippett and Dr Lucy Gates. Full details of the local recruiting sites are listed at https://www.diabetesgenes.org/current-research/startright/startright-recruiting-sites/.

## Abbreviations

BDD: Better Diabetes Diagnosis
BOX: Barts Oxford Family Study
OR: odds ratio
SNP: single nucleotide polymorphism
T1DGC: Type 1 Diabetes Genetic Consortium
T1D-GRS2: type 1 diabetes genetic risk score
TrialNet PTP: TrialNet Pathway to Prevention
